# Surface Wave Multipath Signals in Near-Field Microwave Imaging

**DOI:** 10.1155/2012/697253

**Published:** 2012-04-10

**Authors:** Paul M. Meaney, Fridon Shubitidze, Margaret W. Fanning, Maciej Kmiec, Neil R. Epstein, Keith D. Paulsen

**Affiliations:** ^1^Thayer School of Engineering, Dartmouth College, Hanover, NH 03755, USA; ^2^Department of Radiology, Dartmouth Medical School, Hanover, NH 03755, USA; ^3^Norris Cotton Cancer Center, Dartmouth-Hitchcock Medical Center, Lebanon, NH 03756, USA

## Abstract

Microwave imaging techniques are prone to signal corruption from unwanted multipath signals. Near-field systems are especially vulnerable because signals can scatter and reflect from structural objects within or on the boundary of the imaging zone. These issues are further exacerbated when surface waves are generated with the potential of propagating along the transmitting and receiving antenna feed lines and other low-loss paths. In this paper, we analyze the contributions of multi-path signals arising from surface wave effects. Specifically, experiments were conducted with a near-field microwave imaging array positioned at variable heights from the floor of a coupling fluid tank. Antenna arrays with different feed line lengths in the fluid were also evaluated. The results show that surface waves corrupt the received signals over the longest transmission distances across the measurement array. However, the surface wave effects can be eliminated provided the feed line lengths are sufficiently long independently of the distance of the transmitting/receiving antenna tips from the imaging tank floor. Theoretical predictions confirm the experimental observations.

## 1. Introduction

**Figure 1:**
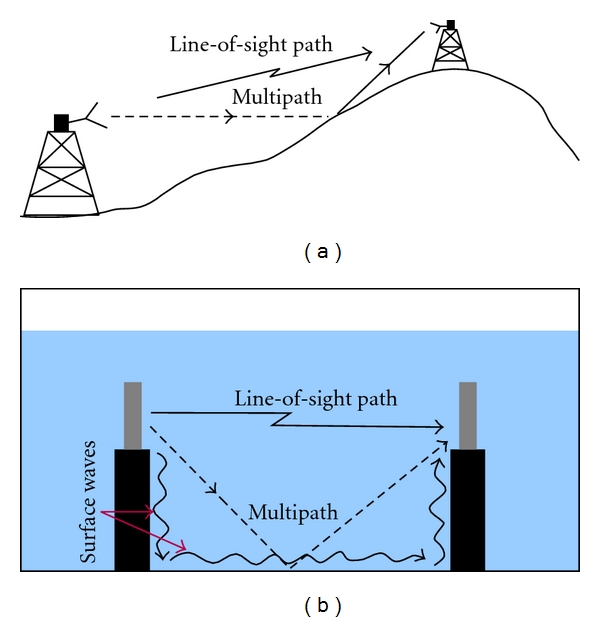
(a) Illustration of a bistatic communication between antenna towers and line-of-site versus possible multipath signals and (b) near-field imaging tank in a liquid medium with possible reflection and surface wave propagation paths.

Multipath signals occur in numerous microwave and RF applications when an unwanted portion of the original transmission propagates along any alternate path and ultimately couples to the receiver distorting the amplitude and phase of the desired signal [1–4]. If the amplitude of the multipath signal is sufficiently large, its impact can be considerable. In multistatic radar and communication systems, these types of interference are most often caused by reflections of either the main beam or side lobes with objects near or actually in the beam path. Classic examples include main beam propagation close to the earth's surface with associated reflections off of the ground or water (Figure 1(a)) [5]. Various approaches can be used to filter or compensate for these reflections through time-gating [6] and signal time synchronization [7].

The potential for interference from multipath signals increases substantially in near-field applications (Figure 1(b)), especially in situations where the receiving and transmitting hardware are integrated. A common form occurs when multiple receive channels are employed with inadequate channel isolation. Commercially available multichannel network analyzers (e.g., ZVT8 by Rohde & Schwarz; Munich, Germany) utilize robust strategies to minimize these signal coupling problems. We are developing multichannel transceiving arrays for medical microwave imaging which exploit near-field concepts to produce electrical property maps (permittivity and conductivity) of tissues of interest [8, 9] and have addressed the issue by incorporating (a) dedicated mixers for each channel, (b) additional solid state switches for isolation, (c) double- and triple-braided coaxial cables, and (d) compartmentalized RF circuitry. The implementation has proven effective for our application achieving channel/signal isolation greater than 130 dB [8]. An alternative data acquisition strategy integrates a commercial, 2-port network analyzer with an electronic switching network to feed an array of antennas [10–12] which is effective but also has limitations because (i) dynamic range is constrained by the provisions of the network analyzer, (ii) two-way signal loss is incurred through the network, and (iii) the switching matrix has relatively poor cross-channel isolation [10].

**Figure 2:**
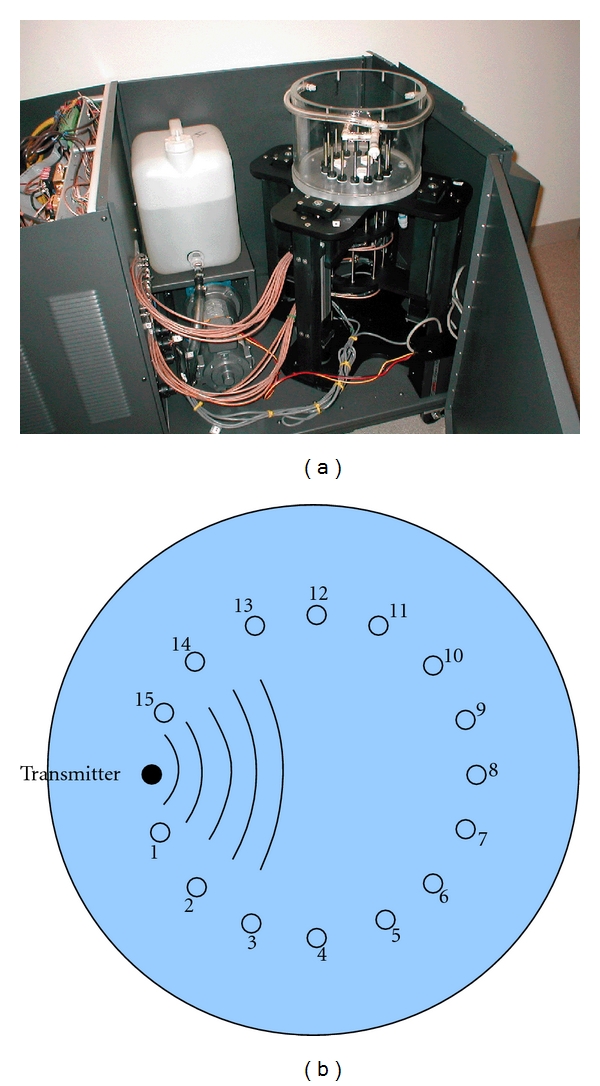
(a) Photograph of the inside of the clinical imaging system showing the imaging tank, liquid reservoir, and antenna motion system. The microwave electronics are housed behind the firewall to the left; (b) schematic diagram of the antenna array configuration.

Equally important in near-field imaging is the multiple paths a signal can take within the imaging zone. Figures 2(a) and 2(b) show a photograph of our clinical breast imaging tank and a schematic diagram of the antenna configuration, respectively. In this situation, the array of 16 monopole antennas surrounds the breast and can be moved to multiple vertical positions. The antennas and target are submerged in a solution of glycerin and water which is lossy over the operating frequency range (700 MHz–3 GHz). Early empirical tests have indicated that reflections off the tank side walls do not impact the desired signals for an array with the antennas mounted on a 15.2 cm diameter circle [13]. Likewise, analysis of the monopole beam patterns as a function of frequency has shown that artifacts are minimal when the array approaches the liquid interface at the top of the tank [14].

With respect to reflections off the bottom surface, the base of the tank was at least 1.8 wavelengths (at the lowest frequency) below the active sections of the antennas in our initial clinical installation when the array was located at its lowest position during an exam. Since minimal multipath signals resulted from reflections off the top liquid surface, symmetry would suggest that the same should be true for the base of the tank (Figure 1(b)). However, surface waves can cause multipath signals that can be especially difficult to eliminate in near-field systems. Their excitation can be complex, but their propagation characteristics along two material interfaces, whether planar or along cylindrically shaped structures, have previously been studied in depth [15–20]. Surface waves can readily propagate at the interface of two dielectric materials or one conductor juxtaposed directly with a dielectric. Their propagation and attenuation characteristics are nominally determined by the electrical properties of the two materials. In addition, their amplitude decays exponentially away from the interface in the perpendicular direction as a function of the lossiness of the complementary materials [15].

It should be noted that these investigations have stemmed partially from our efforts to perform microwave tomographic images on patients in an actual MR scanner for the purpose of exploiting the refined spatial resolution of the MR along with the more specific nature of the tissue dielectric properties. The MR bore is quite small and places a significant constraint of space for the microwave system. Initial attempts included shortening the antenna feed lines associated with the shorter illumination tank. This was where we first encountered multipath signal corruption which subsequently led to this study.

In the following sections, we discuss the theoretical underpinnings of these modes for the geometries present in our system. We demonstrate cases from our current imaging system, where the measurements indicate corruption of the desired signals from multipath signals associated with the base of the tank. We then show experiments that allow us to partially isolate the effects to surface waves propagating along other pathways. We realize that there are a number of propagation modes around the antennas, their feedlines and the tank surfaces, of which the surface waves are only one possible contributor, but understanding these contributions is important. We present an initial strategy for minimizing the effects of these signals which may be instructive for designing other near-field imaging systems, including simulations confirming the earlier theoretical discussion and validating our feedline design strategy.

## 2. Methods

### 2.1. MultiPath Corruption

A challenging situation occurs when a portion of the transmitted signal propagates along an unwanted path and recombines with the original signal at the receiver. Because both occur at the same frequency, filtering is generally ineffective. Time-gating strategies can sometimes be effective when the nature of the multipath signal is well understood [1, 2]. Attenuation is another means of dealing with these unwanted signals. The potential influence of a multipath signal when the original transmission is a continuous wave can be written as

(1) $$\text{Resultant}\;\text{Signal}=A_{\text{comb}}\cos\left(\omega t+\phi_{\text{comb}}\right),$$

where

(2) $$\begin{array}{c} \phi_{\text{comb}}=A_{\text{comb}}\tan^{-1}\left(\frac{B_{1}}{A_{1}}\right),\\ A_{\text{comb}}=\sqrt{A_{1}^{2}+B_{1}^{2}},\\ A_{1}=\left[A_{\text{de}}\cos_{\text{de}}\phi +A_{\text{mp}}\cos\phi_{\text{mp}}\right],\\ B_{1}=\left[A_{\text{de}}\sin_{\text{de}}\phi +A_{\text{mp}}\sin\phi_{\text{mp}}\right]. \end{array}$$

Here, *A*_de_, *A*_mp_, *ϕ*_de_, and *ϕ*_mp_ are the desired and multipath signal amplitudes and phases, respectively, *ω* is the operating frequency, and *t* is time. For example, if the magnitude of the multipath signal is 25 dB below that of the desired signal, the maximum possible amplitude and phase errors in the resultant signal would be 0.48 dB and 3.22°, respectively. For a −15 dB multipath signal, these values increase to 1.42 dB and 10.24°. Clearly, the resultant phase and amplitude errors can become very significant for multipath signals that are on the same order of magnitude as the desired signals. It should be noted that there can be many multipath contributions with a range of amplitude and phase contributions. This single contributor analysis serves to give a flavor that the unwanted effects can be significant and is generalizable to multiple sources.

### 2.2. Surface Wave Analysis

#### 2.2.1. Planar Interface between Plexiglas Base and Coupling Liquid

**Figure 3:**
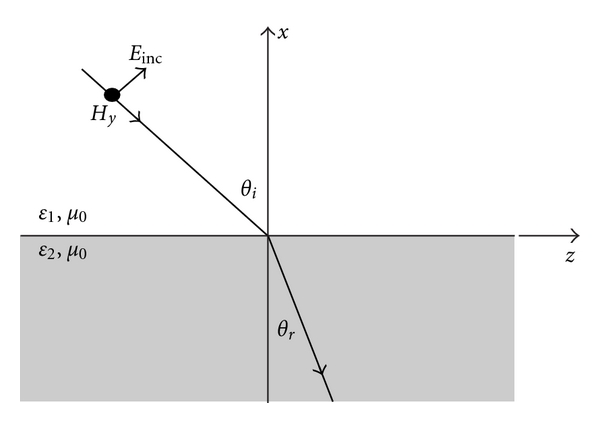
Diagram of a plane wave incident on a planar interface between two dielectric materials.

The first surface mode to be considered involves propagation along the interface between the tank base and coupling liquid. Following the analysis by Stratton [15], Figure 3 shows a plane wave in Region 1 impinging on an interface (*x* = 0) with Region 2. In this case, the magnetic field (*H*_*y*_) is only oriented in the *y*-direction (out of the page). The complex relative dielectric properties in the two regions are *ε*_1_ = *ε*_1_′ − *jε*_1_′′ and *ε*_2_ = *ε*_2_′ − *jε*_2_′′, respectively. The classic surface wave solution occurs when the reflection coefficient is zero (at the Brewster angle), which is complex-valued in this instance. If *A* is the amplitude of the incident plane wave, then the magnetic component of the incident and transmitted waves can be represented as

(3) $$\begin{array}{c} H_{y}=Ae^{\left[jh_{1}x-j\beta z\right]},\;x>0,\\ H_{y}=Ae^{\left[jh_{1}x-j\beta z\right]},\;x<0, \end{array}$$

where *h*_1_^2^ + *β* ^2^ = *k*_1_^2^ and *h*_2_^2^ + *β* ^2^ = *k*_2_^2^ are needed to satisfy the wave equation, *k*_1_ and *k*_2_ are the wave numbers for the two regions, and *β* is the propagation constant. 

The wave impedances for the two regions are given by

(4) $$\begin{array}{c} \begin{array}{c} {\overset{̅}{Z}}_{1}=\frac{h_{1}}{k_{1}}Z_{1}=\frac{h_{1}}{k_{1}}Z_{0}\frac{1}{\sqrt{\varepsilon_{1}^{\prime }-j\varepsilon_{1}^{\prime \prime }}}, \end{array}\\ \begin{array}{c} {\overset{̅}{Z}}_{2}=\frac{h_{2}}{k_{2}}Z_{2}=\frac{h_{2}}{k_{2}}Z_{0}\frac{1}{\sqrt{\varepsilon_{2}^{\prime }-j\varepsilon_{2}^{\prime \prime }}}, \end{array} \end{array}$$

where ${\overset{̅}{Z}}_{1}$ and ${\overset{̅}{Z}}_{2}$ are the free space impedances in the corresponding regions, and ${\overset{̅}{Z}}_{1}$ and ${\overset{̅}{Z}}_{2}$ are the associated wave impedances [21, 22]. This yields

(5) $$\begin{array}{c} h_{1}\kappa =h_{2}, \end{array}$$

where

(6) $$\begin{array}{c} \kappa =\frac{\varepsilon_{2}}{\varepsilon_{1}}=\frac{\varepsilon_{2}^{\prime }-j\varepsilon_{2}^{\prime \prime }}{\varepsilon_{1}^{\prime }-j\varepsilon_{1}^{\prime \prime }} \end{array}.$$

From these relationships, we can solve for

(7) $$\begin{array}{c} h_{1}=k_{0}\sqrt{\frac{\varepsilon_{1}\left(1-\kappa \right)}{1-\kappa^{2}}},\\ h_{2}=k_{0}\sqrt{\frac{\varepsilon_{1}\kappa^{2}\left(1-\kappa \right)}{1-\kappa^{2}},}\\ \beta =k_{0}\sqrt{\frac{\varepsilon_{1}\kappa \left(1-\kappa \right)}{1-\kappa^{2}}}. \end{array}$$

#### 2.2.2. Metallic Coaxial Conductor Surrounded by the Coupling Liquid

**Figure 4:**
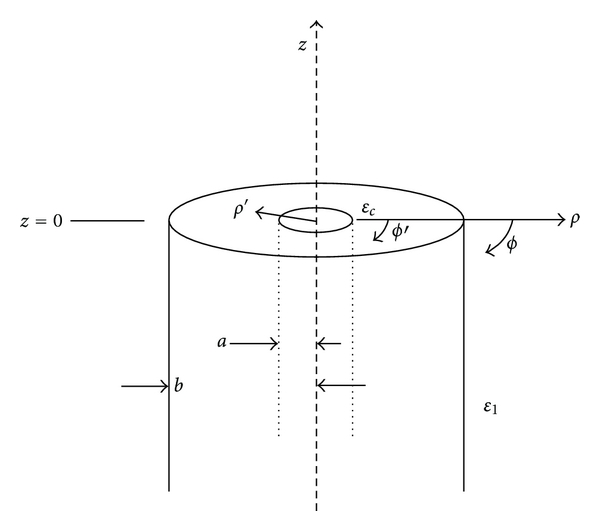
Illustration of the excitation of coaxial surface waves and associated coordinates.

In this situation, we are primarily interested in surface waves propagating along the outside of a coaxial cable, and their associated attenuation as a function of distance after the mode has been sufficiently established. For this analysis, we will consider the case of a coaxial line which is abruptly terminated by an open end (Figure 4).

A coaxial cable supports a TEM-mode electromagnetic field which is incident on the cable opening. Part of the signal is partially reflected into the cable, while a second portion is transmitted as a surface wave propagating along the outside of the surrounding cable. The fields transmitted into the surrounding space can be determined from the distribution at the coaxial opening which can be found by solving the integral equation for the radial component of the electric field over the opening:

(8) $$\begin{array}{cc} & \frac{1}{4\pi \rho }+j\omega \varepsilon_{c}\overset{b}{\underset{a}{\int }}E_{\rho }\left(\rho^{\prime },0\right)K_{c}\left(\rho ,\rho^{\prime }\right)\rho^{\prime }d\rho^{\prime }\\ & \;=\frac{j\omega \varepsilon_{1}}{\pi }\overset{b}{\underset{a}{\int }}E_{\rho }\left(\rho^{\prime },0\right)K_{c}\left(\rho ,\rho^{\prime }\right)\rho^{\prime }d\rho^{\prime }\overset{\pi }{\underset{0}{\int }}\cos\phi^{\prime }\frac{e^{-jkr}}{r}d\phi^{\prime }, \end{array}$$

where *ε*_*c*_ and *ε*_1_ are the complex-valued permittivity of the coaxial cable insulator and the surrounding dielectric materials, respectively, *a* and *b* are inner and outer coaxial radii, respectively, *ω* is the operating frequency in radians, and *k* is the wavenumber in the coupling liquid, where *μ*_0_ is the free space magnetic permeability. *ρ*′ and *ρ* are the radial cylindrical coordinates within and outside of the coaxial cable, respectively, *ϕ*′ is the angular coordinate within the coaxial cable, and *r* is defined as $r=\sqrt{\rho^{2}+{\rho^{\prime }}^{2}-2\rho \rho^{\prime }\cos\phi^{\prime }}$. The variable *K*_*c*_(*ρ*, *ρ*′) is represented as

(9) $$K_{c}\left(\rho ,\rho^{\prime }\right)=j\overset{\infty }{\underset{n=0}{\sum }}\frac{\varphi_{n}\left(\rho \right)\varphi_{n}\left(\rho^{\prime }\right)}{A_{n}^{2}\beta_{n}},$$

where

(10) $$\begin{array}{c} \varphi_{n}\left(\rho \right)=Y_{0}\left(\gamma_{n}a\right)J_{1}\left(\gamma_{n}\rho \right)-J_{0}\left(\gamma_{n}a\right)Y_{1}\left(\gamma_{n}\rho \right),\\ \beta_{n}=\{\begin{array}{cc} \sqrt{k_{c}^{2}-\gamma_{n}^{2}}, & k_{c}>\gamma_{n}\\ \pm j\sqrt{\gamma_{n}^{2}-k_{c}^{2}}, & k_{c}>\gamma_{n}, \end{array}\\ A_{n}^{2}=\frac{2}{\pi^{2}\gamma_{n}^{2}}\left[\frac{J_{0}^{2}\left(\gamma_{n}a\right)}{J_{0}^{2}\left(\gamma_{n}b\right)}-1\right],\;n>0,\\ A_{0}^{2}=\ln\frac{b}{a}. \end{array}$$

The eigenvalue *γ*_*n*_ are solutions of the characteristic equation:

(11) $$Y_{0}\left(\gamma_{n}a\right)J_{1}\left(\gamma_{n}b\right)=J_{0}\left(\gamma_{n}a\right)Y_{1}\left(\gamma_{n}b\right),$$

where *J*_*n*_ and *Y*_*n*_ are Bessel functions of the first and second kind of order *n*, respectively, and *k*_*c*_ is the wavenumber inside the coaxial line. Once *E*_*ρ*_(*ρ*′, *z* = 0) is determined, then electric and magnetic fields can be found at any point (*ρ*, *z*) in the surrounding medium from

(12) $$\begin{array}{c} E_{\rho }\left(\rho ,z\right)=\frac{1}{\pi }\overset{b}{\underset{a}{\int }}E_{\rho }\left(\rho^{\prime },0\right)\rho^{\prime }\overset{\pi }{\underset{0}{\int }}\left(jk+\frac{1}{R^{\prime }}\right)\;z\cos\phi^{\prime }\frac{e^{-jkR^{\prime }}}{R^{\prime }}d\phi^{\prime }d\rho^{\prime }, \end{array}$$

or

(13) $$\begin{array}{cc} E_{\rho }\left(\rho ,z\right) & =\frac{1}{\pi }\overset{b}{\underset{a}{\int }}E_{\rho }\left(\rho^{\prime },0\right)\rho^{\prime }\\ & \;\times \overset{\pi }{\underset{0}{\int }}\left[\frac{1}{\rho }-\left(jk+\frac{1}{R^{\prime }}\right)\right]\frac{\rho -\rho^{\prime }\cos\phi^{\prime }}{R^{\prime }}\frac{e^{-jkR^{\prime }}}{R^{\prime }}d\phi^{\prime }d\rho^{\prime }\\ H_{\phi }\left(\rho ,z\right) & =\frac{j\omega \varepsilon_{1}}{\pi }\overset{b}{\underset{a}{\int }}E_{\rho }\left(\rho^{\prime },0\right)\rho^{\prime }\\ & \;\times \overset{\pi }{\underset{0}{\int }}\cos\phi^{\prime }\frac{e^{-jkR^{\prime }}}{R^{\prime }}d\phi^{\prime }d\rho^{\prime }, \end{array}$$

where

(14) $$R^{\prime }=\sqrt{z^{2}+\rho^{2}+{\rho^{\prime }}^{2}-2\rho \rho^{\prime }\cos\phi^{\prime }}.$$

From these equations it follows that the electromagnetic fields inside the surrounding medium decay approximately as *e* ^−*jkR*′^.

### 2.3. Breast Imaging System

Figure 2(a) shows the illumination tank used in our current clinical breast imaging system. Each monopole antenna consists of an exposed length (3.8 cm) of 2.2 mm diameter semirigid coaxial cable with only the center conductor and insulating Teflon layer intact. For mechanical robustness, the coaxial feed line is enclosed in a 6.4 mm diameter rigid stainless steel tube, and the active section of the antenna is covered with an accompanying length of a Delrin cylinder acting as a protective radome. The space between the copper coaxial outer conductor and the stainless steel sleeve is sealed at either end with silver epoxy to eliminate wave propagation along the gap. The antennas have a nominal return loss of –10 dB over the bandwidth of 700–3000 MHz. The black Delrin fittings at the antenna/tank base contain hydraulic seals through which the antenna feeds pass to allow vertical motion of the array while eliminating any coupling fluid leakage. The 16 antennas are positioned on a 15.2 cm diameter circle, and both sets of 8 interleaved antennas are supported by individual mounting plates under the tank which provide independent motion of the array in groups of 8. The tank is fabricated out of Plexiglas with an inner wall diameter of 27.3 cm and thickness of 1.3 cm, and the base has a thickness of 2.5 cm. All connecting cables are double-braided to minimize stray radiation. In these experiments, the antennas were positioned at heights close to the tank base (that were not used in any clinical exams) to study the multipath phenomenon in detail.

### 2.4. Experimental Imaging Tanks

**Figure 5:**
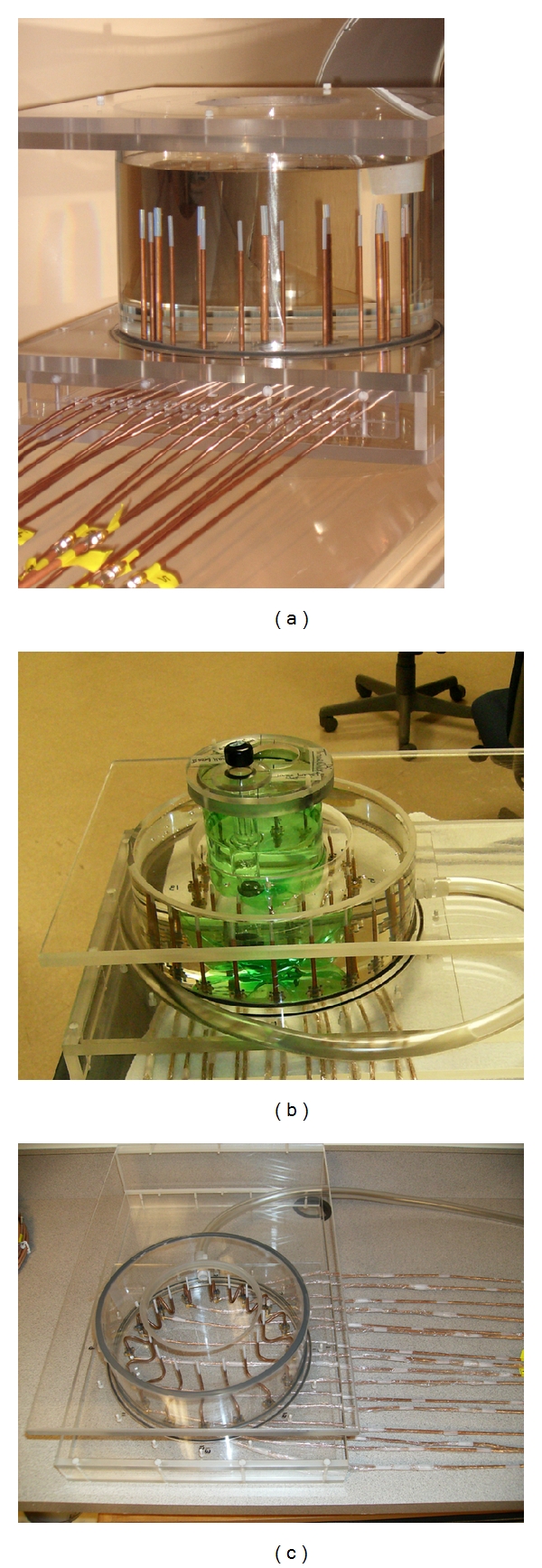
Photographs of the three imaging tanks and antennas used in experiments: (a) taller tank with straight 10 cm feed line lengths, (b) shorter tank with the straight 5 cm feed line lengths, and (c) the short tank with the 10 cm feed line lengths in a serpentine shape.

Figures 5(a), 5(b), and 5(c) show three illumination tanks with different heights, arrays of monopole antennas, and coaxial feed lines that were fabricated from the same Plexiglas and had identical diameters and wall/base thicknesses as Figure 2(a) tank. The feed lines passed through holes in the base of each tank and were fastened to SMA flange connectors which were bolted to the tank floor. The tanks were filled with an 80 : 20 glycerin/water mixture with the liquid level 1.5 cm above the antenna tips. In Figures 5(a) and 5(c), the feed lines were both 10 cm long; however, the latter was bent in a serpentine shape such that the top height of the feed line was only 5 cm above the tank floor. The feed line in Figure 5(b) was straight and was only 5 cm long.

### 2.5. Material Dielectric Properties

**Figure 6:**
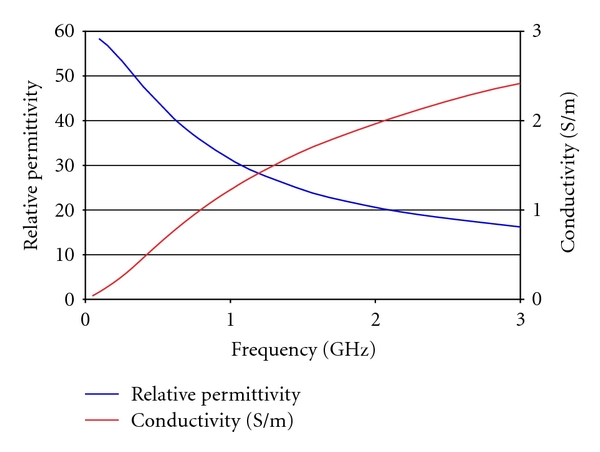
Dielectric properties of the 80 : 20 glycerin : water bath.

In these experiments, we used Plexiglas for the tank materials with a dielectric constant of *ε*_*r*_ = 2.7 that was effectively lossless in this frequency range [23]. The dielectric properties of the 80 : 20 glycerin/water bath are plotted in Figure 6 as a function of frequency.

## 3. Results and Discussion

### 3.1. Clinical System Experiment

**Figure 7:**
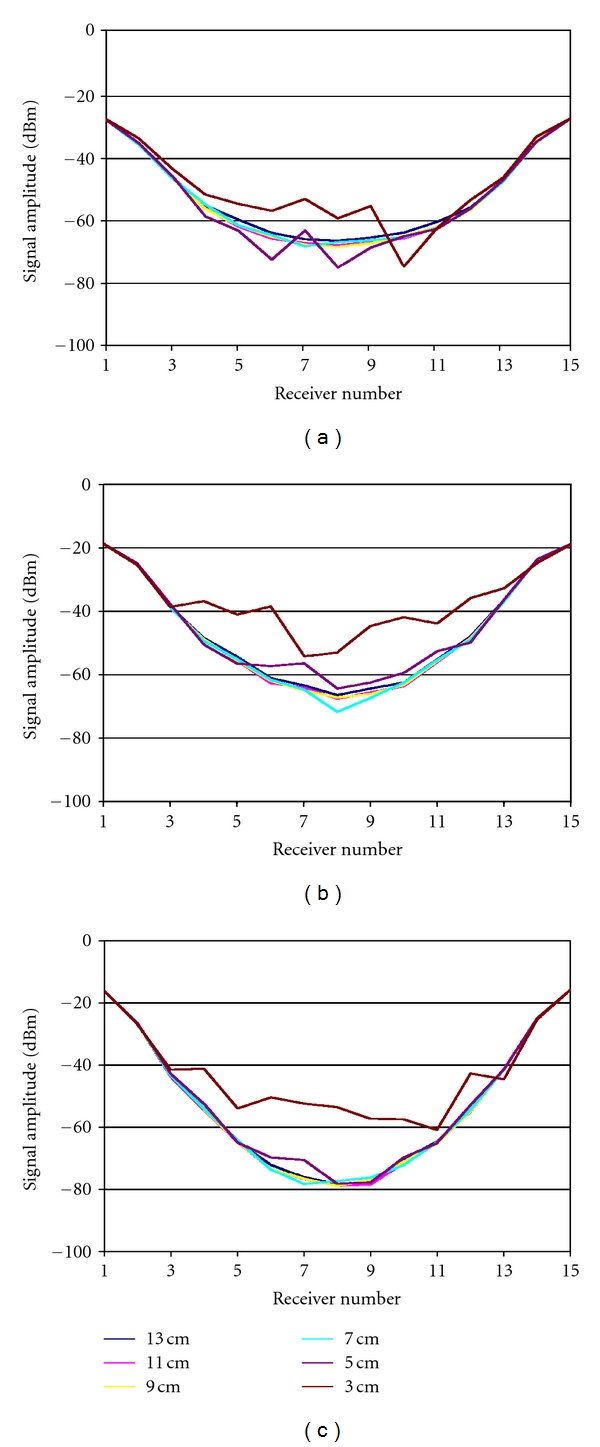
Signal amplitudes measured at the receivers with the clinical system in Figure 2 for a single transmitter over a range of antenna heights above the tank base (3, 5, 7, 9, 11, and 13 cm) for (a) 900, (b) 1300, and (c) 1700 MHz, respectively.

Utilizing the clinical system described in Section 2.2, a +5 dBm signal was transmitted at multiple frequencies over the 700–2500 MHz range from a single antenna and received at the remaining 15 antennas. This sequence was repeated for the array positioned at multiple heights above the tank base. Receive antenna amplitudes are plotted for representative frequencies in Figure 7. At 900 MHz, the measured levels are high for the receivers closest to the transmitter (relative receiver numbers 1, 2, 14, and 15) compared to the rest of the array and do not change dramatically with changes in antenna height. However, the amplitudes are considerably lower for the more distant receive antennas. For antenna heights 7 cm or greater (above the tank floor), the attenuation follows a smooth curve hitting a maximum at antenna 8 (which is furthest away from the transmitter being located on the opposite side of the array). At antenna heights 5 cm and lower, the signal levels begin to deviate from this smooth pattern. The behavior is consistent with the three frequencies shown in Figure 7. Given that the distance from the antennas to the tank side walls did not change during the experiments, and that the antennas were sufficiently far from the liquid/air interface at all times for any array heights (10 cm in the worst case), the corruption of these most distantly received signals appears to be caused by multipath propagation associated with antenna tip proximity to the base of the tank most probably due to reflections off of the tank base or surface wave propagation along the dielectric interfaces.

### 3.2. Experimental Tanks

**Figure 8:**
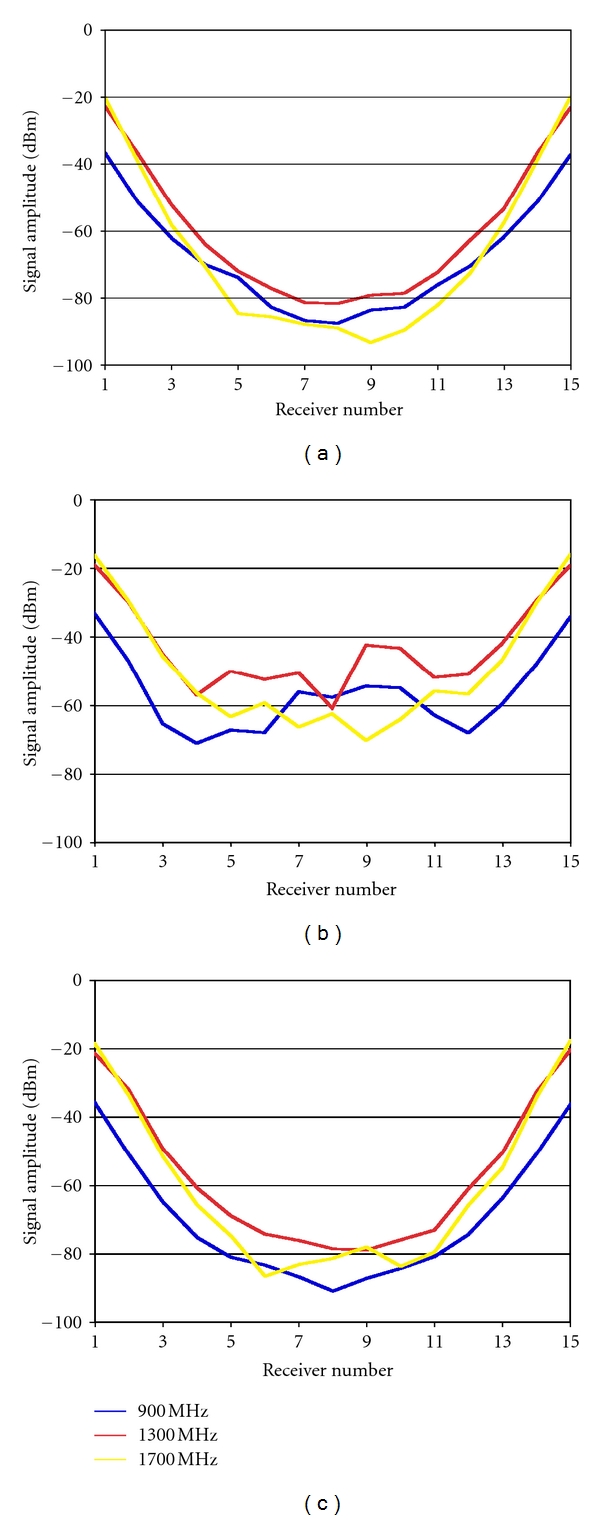
Signal amplitudes measured at the receivers for a single transmitter in the experimental imaging tanks in Figure 5 at 900, 1300, and 1700 MHz with the (a) 10 cm straight, (b) 5 cm straight, and (c) 10 cm curved coaxial feedlines, respectively.

In this set of experiments, we utilized the illumination tanks and antennas described in Section 2.3. Figures 8(a) and 8(b) show the received signals for a single transmitter over a range of frequencies for straight feed line lengths of 10 cm and 5 cm (tanks in Figures 5(a) and 5(b)), respectively. For the longer (10 cm) lines in Figure 8(a), the field patterns appear well-behaved like those in the previous section, when the array was positioned at the largest heights above the tank floor. However, the patterns for the shorter (5 cm) line lengths in Figure 8(b) exhibit corruption of the signals resulting from the longer propagation distances similarly to when the array heights were closest to the base of the tank for the clinical system (in Figure 7). These corrupted signals are nominally between −50 to −70 dBm and occur in uneven patterns relative to the same signals from the longer feed lines which reach −80 to −90 dBm at the furthest antenna. For the shorter propagation distances (i.e., the signals received at antennas 1–4 and 12–15), the attenuation patterns from the shorter and longer feed lines are similar (Figure 8(b) versus 8(a)). These results suggest that the unwanted multipath signal effect is the same in both the experimental tanks and the clinical system tests in the previous section and depends on the antenna height from the base of the tank. However, in Figure 8(c), the antenna feed length is exactly the same as in Figure 8(a), but is curved such that the antenna tip is the same height above the tank floor as the antennas in Figure 8(b) (which led to signal corruption); yet, the measurement results emulate those in Figure 8(a) (which are not corrupted). Here, the active part of the antennas is still positioned on the same 15.2 cm diameter circle as in the other two tanks. These findings show that the principle signal corruption observed in Figure 8(b) is not due to reflections off the tank floor; otherwise it would have appeared in Figure 8(c) since the antennas are at the same position above the base for both Figures 8(b) and 8(c). It should be noted that the signal disruptions for this situation appear more substantial than that for the antennas used in the clinical system as discussed in Section 3.1. This is very likely due to differences in the designs of the hydraulic seals, feedline shielding, and antenna radome for the other system.

**Figure 9:**
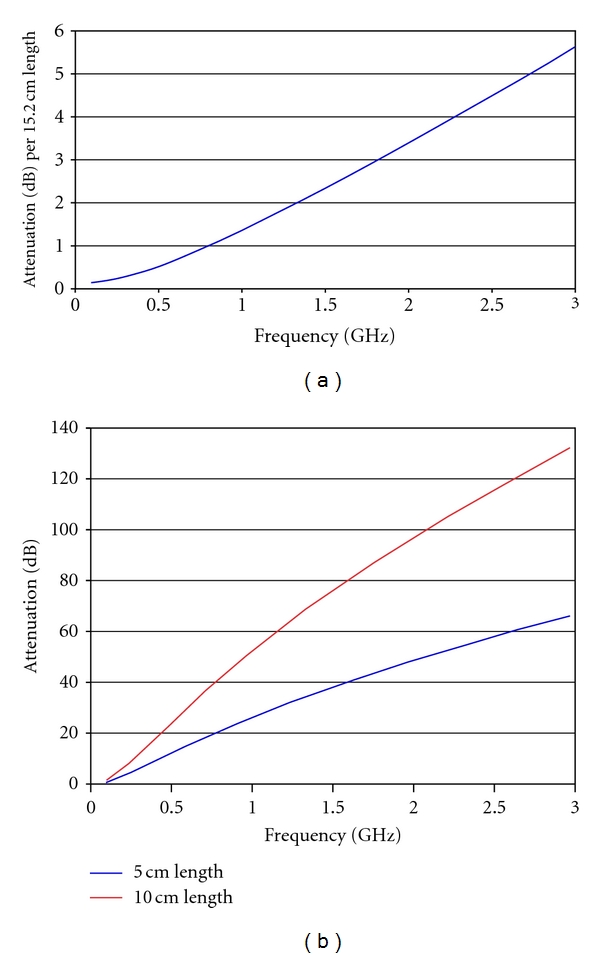
Surface wave attenuation as a function of frequency for (a) the planar mode at the interface between Plexiglas and the bath for a 15.2 cm length and (b) the coaxial mode with a lossy dielectric (80 : 20 glycerin : water bath) surrounding a metal conductor for 5 and 10 cm lengths.

More likely, the multipath signals in Figure 8(b) (with short feed lines) result from surface waves traveling along the outside of the coaxial lines, across the Plexiglas/liquid and/or Plexiglas/air interface and back up the outside of the receiver coaxial feed. The theoretical considerations in Section 2.2 indicate that the attenuation along the coaxial lines is far more substantial than from the planar tank-base surface wave modes.  Figure 9(a) shows a plot of attenuation as a function of frequency for 15.2 cm of Plexiglas/liquid surface waves and indicates that very little attenuation of a surface wave propagating along this interface occurs at the frequencies we used. Thus, in our experiments, the real source of surface wave attenuation comes from propagation along the outside of the coaxial lines.  Figure 9(b) shows plots of the attenuation that results from single 5 and 10 cm lengths of feed line in the coupling fluid. The theoretical predictions of attenuation along the two 5 cm antenna feed lengths (transmit and receive) and the path along the Plexiglas base/liquid interface are approximately 70 dB ({2 × 34 dB} + 2 dB) for the 1 GHz case (values interpolated from Figures 9(a) and 9(b)). Given a transmit power level of +5 dBm, the resultant −65 dBm multipath signal for the shorter line would easily corrupt the desired −81 dBm signal (Figure 8(a)). The 140 dB attenuation for the longer lines easily solves this problem. Only when the feed lines are nearly doubled in length, and the associated surface wave attenuation increased accordingly is the corruption of the desired signals reduced to an acceptable level.

### 3.3. Simulated Field Distributions

**Figure 10:**
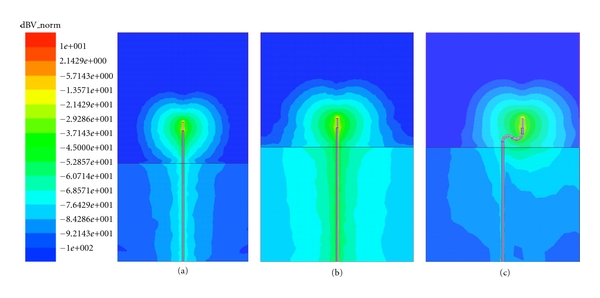
Simulated 900 MHz electric field magnitude distributions (in dB and normalized with respect to the field at the antenna tip) for the (a) long, straight coaxial feed, (b) short, straight coaxial feed, and (c) the longer, serpentine structure.

Along with the analytical discussion in Sections 2.1 and 2.2, we have also performed simulations of the configurations described in Section 2.4. Figures 10(a), 10(b), and 10(c) show the 900 MHz electric field magnitude distributions for the long and short, straight antenna feed and the longer, serpentine structure, respectively. These simulations were computed using ANSYS (Burlington, MA, USA) HFSS version 13.0. For all cases, there is a reasonably broad antenna pattern emanating outwards from the active part of the antennas, and this feature is reasonably similar for all feed line types. For the straight feeds—especially the longer one, it is clear that there are considerable surface currents generated along the coaxial lines. For the shorter straight one, there is a high degree of field strength along the coaxial line within the Plexiglas volume below the horizontal interface. The fields along the interface generally agree with our previous notion that the surface waves preferentially propagate within the lower-loss medium (in this case the Plexiglas) as can be seen by the substantially greater amplitudes directly under the line.

**Figure 11:**
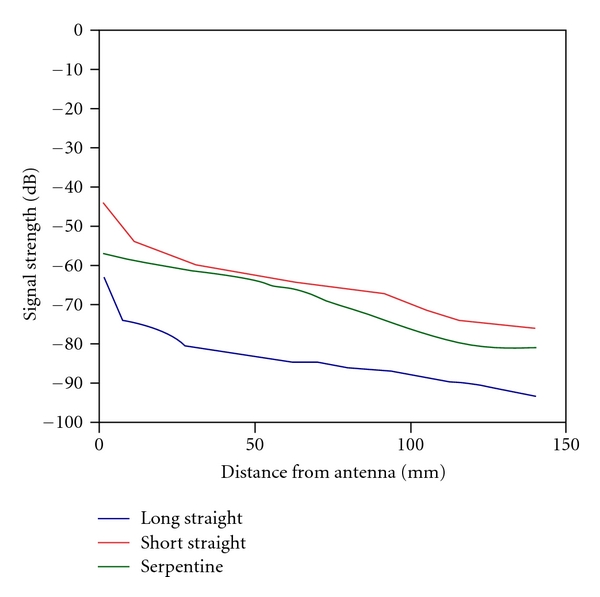
Graph of the 900 MHz electric field magnitudes (dB) along the lower side of the Plexiglas : liquid interface from the point directly under the antenna and extending horizontally for all three distributions shown in Figure 10.

The results for the serpentine feedlines are consistent with previous results in that the field strength in the Plexiglas is lower than that for the short, straight line case.  Figure 11 shows a plot of the field strength just below the liquid interface from a point directly underneath the antenna extending 140 cm to the right for all three corresponding plots. The field values are considerably less for the longer straight line but are also less for the serpentine cases compared to the short, straight cases. For the serpentine case, there seems to be some signal coupling between the lower feedline bend and the Plexiglas. As discussed in Section 1, surface waves do decay exponentially from the surfaces, and given the proximity of the feedlines and the liquid/Plexiglas interface, some coupling is expected.

## 4. Conclusion

The potentially debilitating effects of unwanted multipath signals is a critical consideration in translating near-field microwave imaging approaches into clinical and commercial systems. For our noncontacting antenna approach, surface waves (relative to signal reflections from the imaging tank walls) appear to cause the biggest effects as they propagate along the outside of the transmitting coaxial line across the illumination tank floor and back up the coaxial feed lines of the receivers. When the imaging tank is deep and the transmitting/receiving antenna tips are sufficiently far above the tank base, the surface wave signals are adequately suppressed relative to the transmissions through tissue. The results presented here indicate that 9-10 cm of distance along the feed line is adequate. However, reducing the tank depth is of interest for practical reasons and is essential in some settings and appears possible because reflections from the floor of the tank are still too small to degrade the measured signals propagating through tissue. Indeed, we found that antenna tip distances as little as 5 cm from the tank floor maintain receiver signal fidelity across the array provided the surface wave contributions are attenuated through an equivalent feedline length approaching 10 cm. These findings are significant because they indicate that the antenna array and imaging tank geometry can be altered substantially by manipulating the shape of the antenna feed line, which can be exploited to ensure sufficient surface wave attenuation. There are certainly other mechanisms for multipath propagation including coupling of fields from the feedlines directly to portions of the breast tissue outside the immediate plane of propagation and are certainly good topics for further investigation.
